# Frequency-hopping along with resolution-turning for fast and enhanced reconstruction in ultrasound tomography

**DOI:** 10.1038/s41598-024-66138-2

**Published:** 2024-07-05

**Authors:** Tran Quang-Huy, Bhisham Sharma, Luong Thi Theu, Duc-Tan Tran, Subrata Chowdhury, Chandran Karthik, Saravanakumar Gurusamy

**Affiliations:** 1 Faculty of Physics, Hanoi Pedagogical University 2, Xuan Hoa Ward, Phuc Yen City, Vinh Phuc Province Vietnam; 2 Centre of Research Impact and Outcome, Chitkara University, Rajpura, Punjab 140401 India; 3 Hoa Binh University, Hanoi, Vietnam; 4 Faculty of Electrical and Electronic Engineering, Phenikaa University, Hanoi, 12116 Vietnam; 5 Department of Computer Science and Engineering, Sreenivasa Institute of Technology and Management Studies (SITAMS), Bangalore, India; 6 Robotics and Automation, Jyothi Engineering College, Thrissur, India; 7 Department of Electrical and Electronics Technology, FDRE Technical and Vocational Training Institute, Addis Ababa, Ethiopia

**Keywords:** Biomedical engineering, Electrical and electronic engineering, Gastrointestinal models, Cardiac device therapy, Cardiovascular biology

## Abstract

The distorted Born iterative (DBI) method is considered to obtain images with high-contrast and resolution. Besides satisfying the Born approximation condition, the frequency-hopping (FH) technique is necessary to gradually update the sound contrast from the first iteration and progress to the actual sound contrast of the imaged object in subsequent iterations. Inspired by the fact that the higher the frequency, the higher the resolution. Because low-frequency allows for low-resolution object imaging, hence for high-resolution imaging requirements, using low-frequency to possess a high-resolution image from the first iteration will be less efficient. For an effective reconstruction, the object’s resolution at low frequencies should be small. And similarly, with high frequencies, the object resolution should be larger. Therefore, in this paper, the FH, and the resolution-turning (RT) technique are proposed to obtain object images with high-contrast and -resolution. The convergence speed in the initial iterations is rapidly achieved by utilizing low frequency in the frequency-turning technique and low image resolution in the resolution-turning technique. It is crucial to ensure accurate object reconstruction for subsequent iterations. The desired spatial resolution is attained by employing high frequency and large image resolution. The resolution-turning distorted Born iterative (RT-DBI) and frequency-hopping distorted Born iterative (FH-DBI) solutions are thoroughly investigated to exploit their best performance. This makes sense because if it is not good to choose the number of iterations for the frequency *f*_1_ in FH-DBI and for the resolution of *N*_1_ × *N*_1_ in RT-DBI, then these solutions give even worse quality than traditional DBI. After that, the RT-FH-DBI integration was investigated in two sub-solutions. We found that the lower frequency *f*_1_ used both before and after the RT would get the best performance. Consequently, compared to the traditional DBI approaches, the normalized error and total runtime for the reconstruction process were dramatically decreased, at 83.6% and 18.6%, respectively. Besides fast and quality imaging, the proposed solution RT-FH-DBI is promised to produce high-contrast and high-resolution object images, aiming at object reconstruction at the biological tissue. The development of 3D imaging and experimental verification will be studied further.

## Introduction

The field of clinical diagnostics has been strongly influenced by the advancements in biomedical imaging technology. The explosive growth of information technology and digital media has brought forth clever and advanced techniques for diagnosis and treatment^1^. Wilhelm Roentgen discovered X-rays in 1885, which marked the birth of biomedical imaging technology. Over the past century, the development of advanced technologies, ranging from radiography to computed tomography (CT), positron emission tomography (PET), ultrasonography, single-photon emission computed tomography (SPECT), magnetic resonance imaging (MRI), and more, has led to significant changes in the clinical medicine field. The effectiveness of non-invasive imaging methods has rapidly evolved alongside advancements in computer science.

Biomedical imaging methods involve capturing images of animal or human body parts to gather data about tissues, structures, or specific characteristics of tissues, bones, and even physiological features by administering specialized substances into the body^2^. Currently, there are various biomedical imaging methods as mentioned above, and the research direction of this study focuses on ultrasound imaging, as it is one of the most widely used and represents the gold standard in essential diagnostic examinations, such as obstetrics and cardiology.

Imaging techniques utilizing sound waves have been extensively applied since the development of sonar technology in 1910. Among the most popular methods, using the principles of sonar, is B-mode imaging^3^. This technique is employed for non-destructive diagnostic imaging in the field of medicine. Anatomical grayscale images are created in a way that obtains data from reflection. The amplitude of the tissues’ echoes’ logarithmic compression envelope determines how bright each pixel is. The pulse-echo principle is employed for spatial localization. This non-destructive and imaging technology employs B-mode imaging. Hence, we can differentiate between various media thanks to the qualitative representation of the alteration of the acoustic impedance function in B-mode images. Spatially resolved images can be generated through the utilization of a transducer array^4^ and a probe for a detector element exhibiting elevated convergence characteristics^5^. Although the image quality may deteriorate due to the variations of the amplitude and the phase^6^, the B-mode imaging is reliable and straightforward. Nevertheless, because of the intrinsically qualitative qualities of the B-mode image, medical diagnostics employing this technology are frequently subjective and rely on the competence of the experts. The initial approach to quantitative information gathering is through B-mode imaging. Over the years, structural analysis of images has been explored using various techniques, such as run-length parameters, entropy, mean, standard deviation, and other first- and second-order parameters)^7–9^, statistical boundary descriptor parameters^10^, and wavelet analysis^11^. These solutions have had limited success and are no longer utilized in biomedical devices because these quantitative parameters are not independent of the imaging system.

Compared to the simple reflection of the acoustic echo, the propagation phenomenon of sound waves is much richer. Some energy is dispersed in all directions when the incoming sound wave meets a heterogeneous medium. To ascertain the distribution of acoustic properties of the scatterer, such as sound speed, acoustic attenuation, density, etc., based on a series of dispersed field measurements, it is necessary to perform the inversion of the wave equation. As a result, acoustic tomograms show quantitative information about the target being investigated. Quantitative ultrasound techniques, specifically ultrasound tomography, are known to provide more useful/quantitative information compared to B-mode imaging^12–14^. Ultrasound tomography operates based on scattering. When an acoustic wave encounters an inhomogeneous medium, an energy portion is scattered in all directions. The receiver transducers capture the scattered waves, providing a set of measurements of the scatter field. The inverse scatter issue involves estimating the distribution of acoustic parameters (namely sound speed, density, and attenuation) to reconstruct the heterogeneous object. With this method, the imaged object can be described in greater detail because, instead of relying on the impedance parameters of the acoustic wave, it utilizes multiple attribute parameters of the wave. Therefore, inverse scattering techniques enable the display of quantitative information connected to the object’s mechanical characteristics.

However, inverse scattering techniques face certain limitations, which is why MRI, nuclear, and X-ray imaging are examples of biomedical imaging modalities that have seen greater success than ultrasound tomography devices^15^. First, methods for inverse scattering encounter issues of convergence when recovering objects with significant variations in sound velocity. Due to this limitation, inverse scattering techniques have mainly been used for breast tissue imaging^16,17^, because breast tissue consists mostly of soft tissues. Several independent works have investigated the imaging of bones^18^, that could expand the potential applications of inverse scattering techniques to some extent. Secondly, to obtain the best quality images, scattered data must be collected from multiple angles ranging from 0 to 360°. This is an additional explanation why most of the research on inverse scattering focuses on breast tissue imaging.

Several machine learning algorithms have been employed in the field of ultrasound tomography. These machine learning algorithms play a crucial role in enhancing the performance and capabilities of ultrasound tomography systems for various diagnostic and imaging tasks. Support vector machines (SVM)^19^ are used for classification tasks in ultrasound tomography. It constructs a hyperplane that maximally separates different classes of tissue based on extracted features. Artificial neural networks (ANN) models^20^, such as multilayer perceptrons or convolutional neural networks, have been used for ultrasound tomography. These models learn complex patterns in the data and can be utilized for tasks like tissue classification or image reconstruction. Random forests (RF)^21^, an ensemble learning method, is proposed to combines multiple decision trees to make predictions. It has been employed in ultrasound tomography for tasks like tissue segmentation and classification. Deep learning techniques^22^, particularly convolutional neural networks (CNNs), have shown promising results in ultrasound tomography. CNNs can automatically learn hierarchical features from ultrasound images, enabling tasks such as tissue segmentation and pathology detection. Genetic algorithms (GA)^23^, a search optimization algorithm, has been utilized in ultrasound tomography for tasks like image reconstruction or parameter optimization. It uses evolutionary principles to iteratively improve the solution. Sparse representation algorithms^24^, such as compressed sensing or dictionary learning, have been used in ultrasound tomography to enhance image reconstruction and improve image quality by exploiting the sparsity of ultrasound signals. However, in this work, we use the Tikhonov regularization based on the least square problem, which is used in the simple machine learning algorithms, namely linear or non-linear regression. Our goal is to enhance the imaging quality and convergence rate of objective function recovery based on frequency matching and interpolation techniques.

High computational complexity is a constraint for inverse scattering-based ultrasound tomography. Nowadays, reducing computational complexity and continuously improving image quality are issues that are mainly focused on modern backscattering techniques. The Born approximation is used in most ultrasound tomography studies. For diffraction tomography, approaches as Born Iterative (BI) and Distorted Born Iterative (DBI) are well-known^25–28^. Both the BI and DBI solutions can deal with structures smaller than the wavelength of incident waves.

In the work^28^, the authors proposed a new algorithm to improve the accuracy of ultrasound tomography images by merging the BI and DBI methods. The DBI method features an iterative update of the Green function in each iteration, leading to a notable advantage in terms of faster convergence speed compared to the BI method. But one drawback is that it is much affected by noise. Within the BI method, the Green function remains unchanged throughout the iteration process, thereby endowing this approach with a substantial advantage in effectively mitigating the impact of noise (i.e. not greatly impacted by noise). However, one drawback is that it has a rather high computational complexity. To take advantage of the advantages of BI and DBI methods and overcome their disadvantages, the authors have proposed a solution to combine BI and DBI methods to improve the quality of ultrasound tomography image recovery. Following this solution, the image creation process consists of two steps: First, perform image restoration using BI method in the first few iterations (the advantage of this step is that the restored image is not affected by noise, whereas, if using DBI method, it will be greatly affected by noise, leading to poor image quality right from the first iteration). Second, perform image recovery using DBI method in the remaining loops (the advantage of this step is to get a solution for faster convergence to minimize the total number of iterations required for the image reconstruction process, while, if using BI method, the convergence speed will be slower, leading to a long image recovery process and significantly increased computational complexity). The number of needed iterations with the BI method during the initial phases is very necessary for the best image recovery process, and to minimize the computational complexity inherent in the BI method.

The BI and DBI techniques are extensively employed in the detection of abnormal breast tumors in females. To facilitate this process, a circular measurement configuration is set up around the target object (in this case, the object is positioned at the measurement system’s center). In theory, the placement of transmitters and receivers on the measurement configuration can follow either an even or random distribution. The quantity of transmitters and receivers is contingent upon the specific scenario, depending on the circumstance and the real needs. Opting for a substantial number of transmitters and receivers results in increased complexity within the measurement system, thereby necessitating substantial computational resources and significant memory capacity to effectively handle and process the voluminous information. Conversely, selecting less transmitters and receivers in the system would correspondingly reduce the computational and memory requirements. The positions of transmitters and receivers can either differ from one another or have the potential to overlap. Nevertheless, owing to advancements in ultrasonic transducer technology, modern transducers have the capability to both emit and capture ultrasonic signals, thereby allowing for the possibility of overlapping positions between transmitters and receivers. Most of the studies on BI and DBI use probes that are spaced evenly on the measurement system and use Tikhonov’s regularization for image recovery^28^. The approach is proposed to minimize the complexity and enhance the quality of the recovered images such as using the *l*_1_- regularized least squares problem instead of the Tikhonov method in addressing the inverse issue^24^. This approach uses a measurement configuration where the probes are randomly arranged on the measuring system instead of a measurement using evenly spaced probes.

The common iterative Born method has two variants: the coarse resolution initial value (CRIV) and the quadric-phase source (QS) approaches^29^. These two algorithms offer accurate recovery results without too much computation overhead for low-contrast subjects. However, the QS algorithm proved ineffective for high-contrast subjects. In the work^12^, it is shown that after increasing the sample density by employing zero paddings, the frequency domain interpolation method can produce good results in recovered quality using bilinear interpolation. The primary significant drawback of this approach is its convergence characteristics. Edge detection^27^ has been proposed to enhance convergence speed and improve recovery quality during the iterative procedure, but there are still disadvantages in complexity and noise sensitivity. To speeding up the recovery process, a multi-level fast multiplexing algorithm (MLFMA)^30^ is applied. However, it is challenging to implement the MLFMA in practice due to high setup costs. RFE feature selection^31^ is considered for breast cancer classification and AI is used in medical imaging informatics^32^.

The resolution-turning technique (interpolation) has been studied and applied to the DBI in the works^33,34^. The interpolation approach is used to construct the bigger matrix *N*_2_ × *N*_2_ after recovering the goal function for the matrix of size *N*_1_ × *N*_1_. This method recovers images with higher quality than the DBI and with a noticeably shorter computation time. In the works^35,36^, frequency-hopping (dual- or multi-frequency) technique has been researched and used with the DBI. The objects in *N*_f1_ and *N*_f2_ loops are recovered using the frequencies *f*_1_ and *f*_2_. The low-frequency *f*_1_ guarantees that the algorithm converges to a contrast level near to the real value but with limited resolution of spatial features. The convergence can be maintained while resolution of spatial is improved using high-frequency *f*_2_. The actual contrast level and the previously predicted contrast level only differ by a minor amount (the Born approximation condition is met). We know that low frequency allows low-resolution objects to be imaged, hence using low frequency to obtain high-resolution images from the first iteration is inefficient for high-resolution imaging requirements. For effective reconstruction, at low frequencies, the resolution of the subject should be small. And similarly, with high frequencies, the object resolution should be larger. Therefore, in this paper, with the aim of imaging objects with high contrast, high resolution, and fast and quality reconstruction, an improved ultrasound tomography method by using the frequency-hopping and resolution-turning technique is proposed. Consequently, the normalized error and total runtime for imaging remarkably decreased in comparison to the DBI approach.

This work has the following structure. To detecting small targets, the distorted Born iterative (DBI) approach, which relies on the principles of inverse scattering, is discussed in Sect. 2. Section 3 describes the proposed method, namely the DBI method exploiting resolution-turning (RT-DBI), frequency-hopping (FH-DBI), and resolution-turning along with frequency-hopping (RT-FH-DBI), respectively. Section 4 presents the simulations results of the RT-DBI, FH-DBI, and RT-FH-DBI, respectively. Section 5 presents the discussion about the proposed method. At last, Sect. 6 summarizes our findings. In this study, the RT-DBI and FH-DBI solutions are carefully examined to identify the effective number of iterations with lesser resolution and lower frequency, respectively (for the RT-DBI, the number of iterations with smaller resolution *N*_1_ × *N*_1_ is *N*_N1_ = 1; and for the FH-DBI, the iteration number with lower frequency *f*_1_ is *N*_f1_ = 2). Then, to provide fast and efficient imaging, the RT-FH-DBI integration was proposed. We noticed that the optimal performance would come from using a lower frequency* f*_1_ both before and after the RT with the number of iterations being *N*_Nf11_ = 1 and *N*_Nf12_ = 1, respectively.

### Distorted born iterative (DBI) method

**Figure 1:**
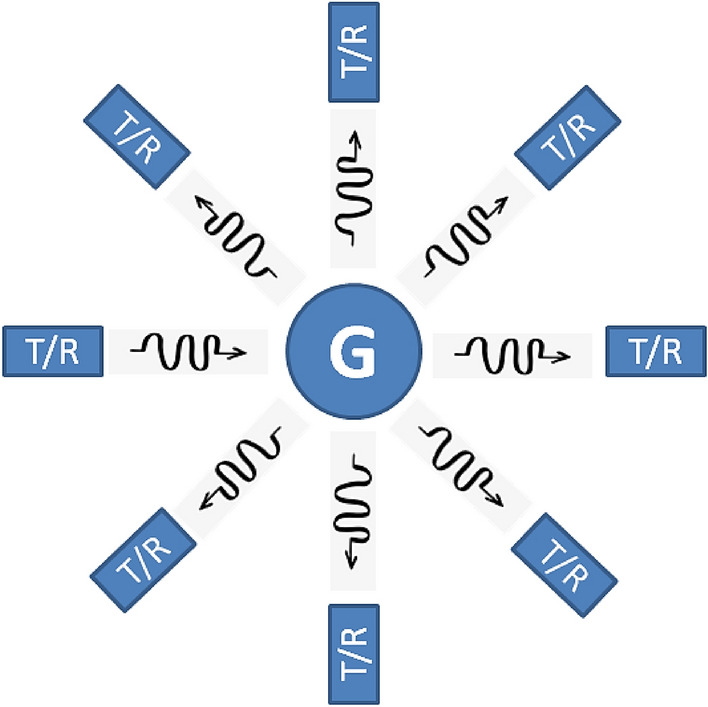
The acoustical and geometrical configuration of the DBI (T/R stands for transmitters and receivers).

Figure 1 depicts how to configure the measurement for T/R probes to obtain dispersion data by placing them in a circle around the object. For a corresponding measured data value at a time, only one transmitter and one receiver are operating. To recover the acoustic contrast of the scattering goal, the DBI is used for this data processing. Any tissue can be detected in this environment using DBI processing the obtained data.

The scenario where an incident wave propagates through a non-uniform medium, such as one containing an anomalous tumor, the impact of the incident wave on the target can give rise to diverse situations. Specifically, the following situations may appear: when the dimensions of the object surpass the incident wave’s wavelength by a substantial margin, the ultrasonic signal will undergo reflection; conversely, when the object’s size is smaller or equivalent to the incident wavelength, the ultrasonic signal will scatter in multiple directions surrounding the target. In case of scattering, if the difference in speed of wave propagation between the background medium and the object medium is small, weak scattering will occur, if the difference is large, then the strong scattering phenomenon will occur.

In the measurement configuration of the DBI method, it is assumed that we have *N*_t_ transmitters and *N*_r_ receivers. Multiple transmitters (*N*_t_) are strategically positioned around the object, each at distinct angles, enabling the transmitter–receiver process to capture comprehensive information about the object. The transmission and reception of ultrasonic signals follow a systematic procedure as outlined below: The first transmitter initially just sends out ultrasonic signals (while the remaining transmitters remain inactive), and all *N*_r_ receivers detect the resulting scattered signals. This results in a collection of measured values that comprise *N*_r_ measurements and correspond to the position of the first transmitter. Subsequently, the second transmitter becomes active, and all receivers capture the scattered signal at the second transmitter’s position, resulting in a second set of measured values (i.e. 2 × *N*_r_ measurements). This iterative process persists until reaching the final transmitter (*N*_t_). As a result of the measurement strategy, there are *N*_t_ sets of obtained values (i.e. *N*_t_ × *N*_r_ measurements). Lastly, by combining *N*_t_ sets of measurements, we can obtain the object from all viewpoints. Consequently, only one transmitter operates at each designated time in accordance with the ultrasonic transceiver procedure described above.

**Figure 2:**
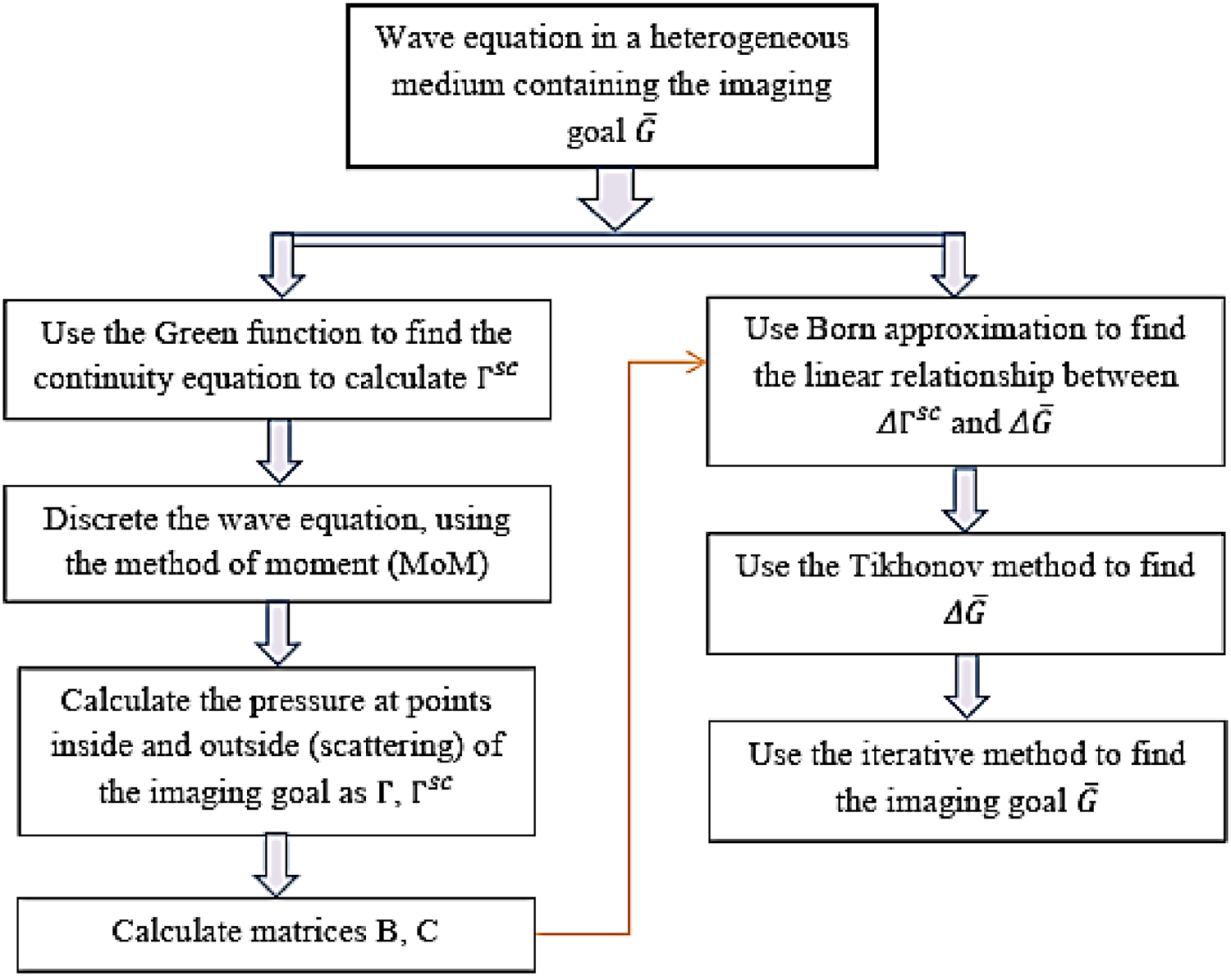
The computational procedure of the DBI method.

The computational procedure of the DBI method is shown in Fig. 2.

The homogeneous medium (M_1_) with a wavenumber of *η*_0_ is considered to exist in limitless space. In this medium, an object (M_2_) with constant density and wavenumber *η*(z) is positioned. The system’s wave equation is written as follows:

1 $$ \nabla^{2} \Gamma \left( {\vec{z}} \right) + \eta_{0}^{2} \Gamma \left( {\vec{z}} \right) = - G\left( {\vec{z}} \right)\Gamma \left( {\vec{z}} \right) $$

where

2 $$G\left(\overrightarrow{z}\right)={\upeta }_{1}^{2}-{\upeta }_{0}^{2}-{\rho (z)}^{1/2}{\nabla }^{2}{\rho (z)}^{-1/2},$$

3 $${\eta }_{1}\left(z\right)=\frac{\omega }{{c}_{1}(z)}+i\alpha \left(z\right).$$

Parameters wavenumber, sound speed, attenuation, density, and angular frequency are denoted as ${\eta }_{1}\left(z\right)$, ${c}_{1}(z)$, $\alpha \left(z\right)$, $\rho (z)$ and *ω*, respectively.

The incident wave is designated as $\Gamma^{inc} \left( z \right)$, and scattering waves can be produced as shown below.

4 $$ \Gamma^{sc} \left( z \right) = \mathop \smallint \limits_{{\Omega }}^{{}} G\left( {z^{\prime}} \right)\Gamma \left( {z^{\prime}} \right)G_{0} \left( {{\upeta }_{0} ,z - z^{\prime}} \right) dz^{\prime} $$

The formula for calculating the total pressure inside the heterogeneous zone Ω is $\Gamma \left( z \right) = \Gamma^{inc} \left( z \right) + \Gamma^{sc} \left( z \right)$, and $G_{0} \left( {{\upeta }_{0} ,z - z^{\prime}} \right)$ is the Green function. In the scenario of homogeneity, *G*_0_ is a first-order zero-order Hankel function.

5 $${G}_{0}\left({\upeta }_{0},z-{z}{\prime}\right)=\frac{-i}{4}{H}_{0}^{\left(1\right)}\left({\upeta }_{0}\left|z-{z}{\prime}\right|\right)=\frac{-i}{4}\sqrt{\frac{2}{\pi {\upeta }_{0}\left|z-{z}{\prime}\right|}}{e}^{i({\upeta }_{0}\left|z-{z}{\prime}\right|-\pi /4)}.$$

The formula for the overall pressure is

6 $$ \Gamma \left( z \right) = \Gamma^{inc} \left( z \right) + \mathop \smallint \limits_{{\Omega }}^{{}} G\left( {z^{\prime}} \right)\Gamma \left( {z^{\prime}} \right)G_{0} \left( {{\upeta }_{0} ,z - z^{\prime}} \right) dz^{\prime} $$

The method of moment (MoM), an effective solution, is used to solve Eq. (6). In vector form with dimensions of *N*^2^ × 1, the mesh point pressure can be calculated as:

7 $$ \overline{\Gamma } = \left( {\overline{I} - \overline{C}.D\left( {\overline{G}} \right)} \right)\Gamma^{inc} . $$

The scatter vector *N*_t_*N*_r_ × 1 is obtained from the exterior points:

8 $$ \overline{\Gamma }^{sc} = \overline{B}.D\left( {\overline{G}} \right). \overline{\Gamma }, $$

in which the operator converts a vector to a diagonal matrix, a matrix with Green *G*_0_(z,z’) coefficients among all pixels, a matrix with Green coefficients *G*_0_(z,z’) every pixel from reaches the receiver, and an identity matrix are denoted by *D*(.), $\overline{B }$, $\overline{C }$, and $\overline{I }$, respectively. In Eqs. (7) and (8), the unknown variables $\overline{\Gamma }$ and $\overline{G}$ are present. The (7) and (8) forward equations are rewritten using the first-Born approximation as follows^37^:

9 $$ \Delta \Gamma^{sc} = \overline{B}.D\left( {\overline{\Gamma }} \right).\Delta \overline{G} = \overline{M}.\Delta \overline{G}, $$

where $\overline{M} = \overline{B}.D\left( {\overline{\Gamma }} \right)$. We will obtain a scalar value $\Delta \Gamma^{sc}$ and a $\overline{M }$ matrix for each transmitter and receiver. It can be remarkable that the amount of pixels in the ROI equals to *N* × *N* variables of an indefinite vector $\overline{G }$.

Iterations are a method for estimating object function:

10 $$ \overline{G}^{n} = \overline{G}^{{\left( {n - 1} \right)}} + \Delta \overline{G}^{{\left( {n - 1} \right)}} , $$

in which steps $\overline{G}^{n}$ and $\overline{G}^{{\left( {n - 1} \right)}}$ represent the previous and current object functions, respectively; solving the Tikhonov regular problem^38^, $\Delta \overline{G}$ can be estimated.

11 $$ \Delta \overline{G} = {\text{arg}}\mathop {\min }\limits_{{\Delta \overline{G}}} \Delta \overline{\Gamma }^{sc}_{t} - \overline{{M_{t} }} \Delta \overline{G}_{2}^{2} + \gamma \Delta \overline{G}_{2}^{2} , $$

in which, $\overline{M}_{t}$ and $\Delta \overline{\Gamma }^{sc}$ are the system matrix of the size ${N}_{t}{N}_{r}\times {N}^{2}$, and the vector of the size ${(N}_{t}{N}_{r}\times (1)$ contains the discrepancy between the scattering signals that were measured and those that were predicted. The parameter for regularization is *γ*. The selection of the regularization parameter necessitates careful consideration due to its significant impact on the stability of the imaging system. An elevated value of *γ* will result in an unrefined reconstructed image. Nevertheless, employing a tiny value of *γ* results in an increased computational complexity. In this study, the selection of the normal parameter *γ* is based on its functional relationship with the forward error. Through the utilization of a Rayleigh quotient iteration, the value *σ*_0_, (i.e. the initial singular value of the inverted matrix *M*_t_) is estimated. Subsequently, the selection of *γ* is determined based on the approach presented in the study^37^. It should be noted that as the inverse matrix *M*_t_ undergoes modifications in each iteration, the value of *γ* is correspondingly adjusted. In our simulation situation, the value of *γ* is computed as 1.2 × 10^−12^ in the first loop.

The computational cost and memory requirement of the Tikhonov method is O(*N*_t_*N*_r_*N*^2^). Even for problems of relatively small size, the memory requirement becomes prohibitively large. Hence, it is imperative to seek solutions that effectively decrease the computational workload and enhance the computational speed for both BI and DBI approaches.

Algorithm 1 presents the DBI procedure. In the DBI method, we can calculate the specific value of each pixel in the region of interest. When there is a small-sized inhomogeneous medium, ultrasound waves will be scattered, and we collect the scattered data. The DBI method allows us to accurately determine the position and shape of the object. We observe that the core issue lies in the algorithm’s ability to precisely reconstruct the object with high efficiency. Due to the aforementioned reasons, during the development of the DBI method, we idealized the model and parameters with the aim of researching and developing an image reconstruction algorithm superior to traditional methods. Consequently, we established the target object for reconstruction as a simple cylindrical shape, and assumed the surrounding environment to be homogeneous (ignoring the presence of breast tissues, etc.). The critical task is to reconstruct the image to closely resemble the target function as much as possible.

In fact, we have verified experimental data for the conventional DBI algorithm, experimental data provided by Bioacoustics Research Lab, University of Illinois at Urbana-Champaign (https://www.brl.uiuc.edu/Projects/inverse_scattering_study.php). Once we had a validated DBI model, we continued to develop the model by integrating frequency combination and interpolation techniques solely using simulation data. While experimental validation is crucial for confirming the effectiveness of ultrasound imaging methods, including DBI, simulation-based development can serve as a valuable tool for methodological exploration and refinement. By leveraging simulation data, we can systematically investigate and optimize the integration of frequency combination and interpolation techniques within the DBI framework, laying the groundwork for subsequent validation with experimental data and eventual translation into clinical practice.

**Algorithm 1:**
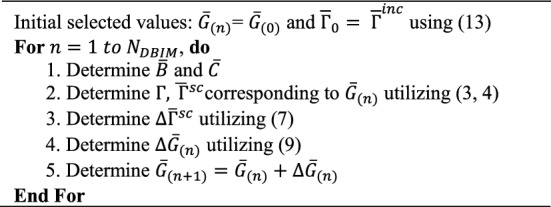
The DBI method.

### The proposed method

In this subsection, to maximize the performance, the RT-DBI and FH-DBI solutions are thoroughly investigated to find the efficient number of iterations with smaller resolution and with lower frequency, respectively. The RT-FH-DBI integration was then looked into in order to provide fast and effective imaging.

### DBI method exploiting resolution-turning (RT-DBI)

In imaging to detect early objects causing breast cancer, the size of the measuring system is determined, so the number of probes that can be arranged on the measuring system can also reach a determined limit. With the need to create images with high resolution, the number of variables is much larger than the number of measurements. If we restore the object right in the first loop with a large number of variables, the estimation is quite less accurate than estimating with a small number of variables (small resolution). Therefore, starting images with a small resolution is necessary to be able to estimate and reach a certain convergence.

It is easier to double the size of the closest neighbor interpolation used as the RT by substituting each pixel with four pixels of the identical value. The final image is bigger than the initial yet retains all the initial information. For interpolation techniques, the simplest and fastest one is the nearest neighbor. There are several complicated interpolation techniques, including bilinear, bicubic, spline, etc. The closest neighbor approach, on the other hand, is chosen since it does not produce novel data values and is time-consuming^39^.

**Figure 3:**
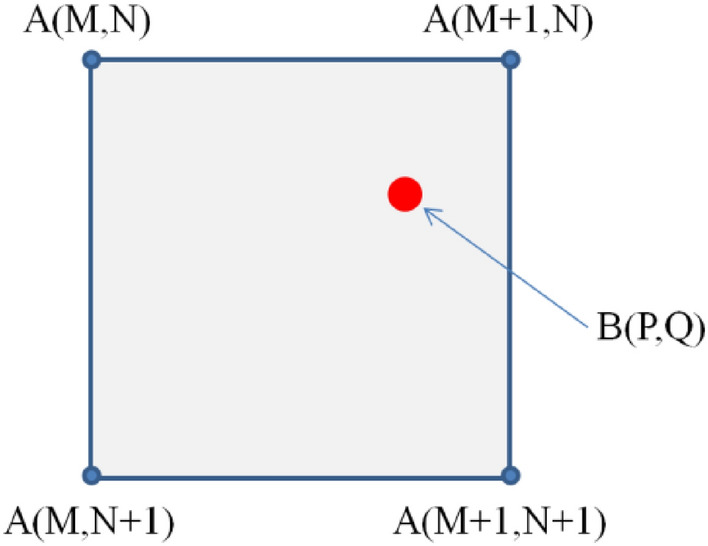
Coordinate system when determining pixels of nearest neighbor interpolation technique.

To make it easier to visualize the nearest neighbor interpolation technique, we consider Fig. 3. The data points in set A represent pixels from the original image (input image), while the data points in set B represent pixels of the output image (interpolated image). So, for each pixel in the output image B, we must compute the nearest neighbor pixels in the original image A. Furthermore, we only need to rely on 4 specific data points: A (M, N), A (M + 1, N), A (M, N + 1), and A (M + 1, N + 1). The execution procedure of the interpolation process is described as: If (Q-N < N + 1-Q), then pixel is one of the top two, and vice versa, pixel is one of the bottom two. If (P-M < M + 1-P), then one of the "left" two is pixel, and vice versa, one of the "right" two is pixel.

The iteration number performed using an area of raw mesh integration sized *N*_1_ × *N*_1_ is represented as *N*_N1_. Accordingly, the iteration number conducted with an integrated area sized *N*_2_ × *N*_2_ is denoted as *N*_N2_ = *N*_sum_−*N*_N1_. Figure 3 shows the RT-DBI deployment process.

Algorithm 2 exposes the RT-DBI procedure.

**Algorithm 2:**
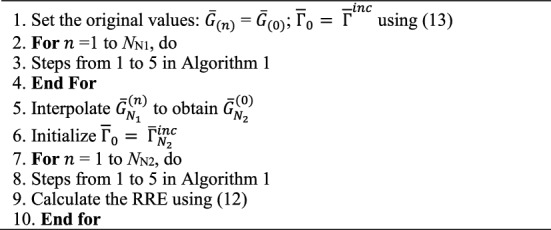
The RT-DBI.

**Figure 4:**
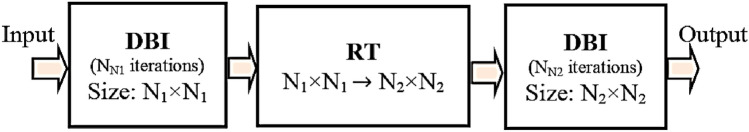
The RT-DBI was performed in stages.

To quantify the effectiveness of the DBI method, the objective function is restored through several iterations. Subsequently, the relative residual error (RRE) is determined and compared with the original image at each iteration. Let $C$ represents the original image with dimensions N × N pixels (i.e., the ideal objective function) and $\widehat{C}$ denotes the restored image. The RRE is calculated as follows (Fig. 4):

12 $$RRE=\sum_{{\varvec{i}}=1}^{{\varvec{N}}}\sum_{{\varvec{j}}=1}^{{\varvec{N}}}\frac{\left|{{\varvec{C}}}_{{\varvec{i}}{\varvec{j}}}-{\widehat{{\varvec{C}}}}_{{\varvec{i}}{\varvec{j}}}\right|}{{{\varvec{C}}}_{{\varvec{i}}{\varvec{j}}}}$$

The RRE is a metric used to assess the quality of image reconstruction by quantifying the discrepancy between the observed data and the data predicted by the reconstructed image. A lower RRE indicates a better reconstruction quality, as the predicted data closely matches the observed data. This suggests that the reconstructed image accurately represents the object being imaged. A higher RRE indicates poorer reconstruction quality, as there is a significant discrepancy between the predicted data and the observed data. This suggests that the reconstructed image does not accurately represent the object. RRE is closely related to the convergence rate in iterative image reconstruction methods, such as the DBI used in ultrasound tomography. The convergence rate refers to how quickly an iterative algorithm approaches the final solution (i.e., the reconstructed image) as the number of iterations increases. The rate at which RRE decreases over iterations can be used to estimate the convergence rate of the algorithm. A rapid decrease in RRE implies a faster convergence rate, while a slow decrease indicates a slower convergence rate.

### DBI method based on frequency-hopping (FH-DBI)

**Figure 5:**
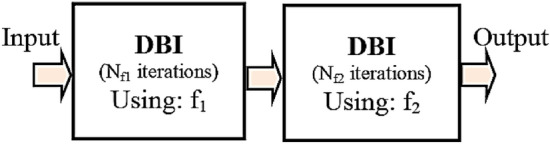
The FH-DBI implementation steps.

The FH approach is used for enhancing resolution, recovery quality, and convergence speed. To estimate the contrast of the ultrasound in the iterations *N*_f1_ and *N*_f2_ (*N*_f2_ = *N*_sum_−*N*_f1_), this approach employs two frequencies f_1_ and f_2_, respectively. Figure 5 shows the FH-DBI deployment procedure.

Algorithm 3 represents the FH-DBI procedure.

**Algorithm 3:**
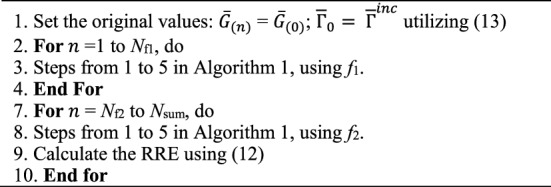
The FH-DBI.

### The proposed DBI method exploiting resolution-turning and frequency-hopping (RT-FH-DBI)

In the frequency-hopping method (FH-DBI), the choice of initial frequency relies on the Born approximation conditions. With the frequency *f*_1_, the initial image is reconstructed with a contrast level *c*_1_. Subsequently, by selecting a frequency step value, we obtain the frequency *f*_2_, and the image is reconstructed with an updated contrast level *c*_2_, derived from the initial contrast *c*_1_. This incremental frequency approach is continued, gradually increasing the frequency, leading to the recovery of images with certain desired contrast level c* (*c*_1_ < *c*_2_ < … < c*). The significance of incrementally increasing the frequency is the progressive enhancement of the image resolution in the reconstruction and the approach towards reconstructing images at the level of biological morphology.

The resolution-turning technique has been applied in DBI (RT-DBI) to improve imaging quality and reduce the total runtime of image reconstruction. There are three steps to this process. Using the DBI, the reconstruction process begins with a raw meshed integration region that is *N*_1_ × *N*_1_ in size. The convergence can be achieved rapidly using *N*_N1_ iterations. The object’s average background value is the outcome at this point. In the subsequent phase, the interpolation from *N*_1_ × *N*_1_ to *N*_2_ × *N*_2_ is applied to the previous stage’s acquired result. Lastly, the DBI is used to continually recreate the second stage result with the necessary size of *N*_2_ × *N*_2_ and *N*_N2_ iterations.

Our contribution to this work is proposing a hybrid solution by combining the resolution-turning and frequency-hopping techniques in the DBI method (RT-FH-DBI). The convergence speed in the initial iterations is rapidly achieved by utilizing low frequency in the frequency-turning technique and low image resolution in the resolution-turning technique. It is crucial to ensure accurate object reconstruction for subsequent iterations. The desired spatial resolution is attained by employing high frequency and large image resolution. This combination efficiently achieves rapid convergence, enhances image quality, and minimizes imaging time.

**Figure 6:**
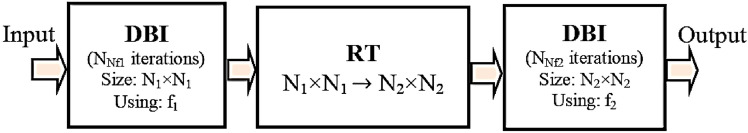
The implementation stages of the first RT-FH-DBI approach (RT-FH-DBI-1).

The RT-FH-DBI is proposed in two approaches: The first and second RT-FH-DBI. Figure 6 exhibits the deployment procedure of the first RT-FH-DBI. Assumed that the amount of iterations performed with a raw mesh integration area of size *N*_1_ × *N*_1_ using *f*_1_ is *N*_Nf1_, the number of iterations performed with an area of size *N*_2_ × *N*_2_ using *f*_2_ is *N*_Nf2_ = *N*_sum_−*N*_Nf1_.

**Figure 7:**
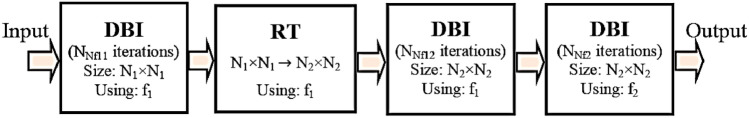
The implementation stages of the second RT-FH-DBI approach (RT-FH-DBI-2).

Figure 7 exhibits the deployment process of the second RT-FH-DBI. Assumed that *N*_Nf11_ represents the iteration number executed with an area of raw mesh integration of the size of *N*_1_ × *N*_1_, using *f*_1_, therefore *N*_Nf12_ represents the iteration number performed with the one that is of the size of *N*_2_ × *N*_2_, using *f*_1_. At last, the iteration number performed with the one that is of the size of *N*_2_ × *N*_2_, using *f*_2_ is *N*_Nf2_ (*N*_Nf2_ = *N*_sum_−*N*_Nf11_−*N*_Nf12_).

Algorithm 4 exposes the RT-FH-DBI-1 procedure.

**Algorithm 4:**
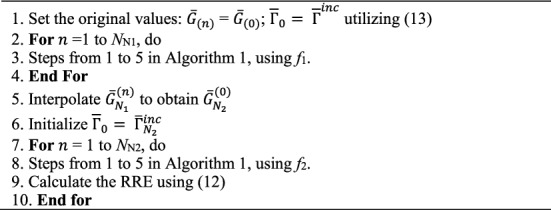
The RT-FH-DBI.

Algorithm 5 shows the RT-FH-DBI-2 procedure.

**Algorithm 5:**
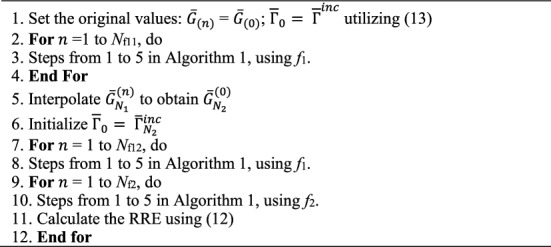
The second RT-FH-DBI.

## Simulations results

The DBI method is widely used in the creation of ultrasound tomography images. Previous studies have also constructed experimental imaging systems to validate the DBI method^37^. Leveraging the experimentally validated DBI method, the goal of our current research is to develop an algorithm for target reconstruction with improvements in convergence speed, image quality, and imaging time.

In the DBI method, it is possible to calculate the specific values for every pixel within the region of interest. The scattering data can be obtained with just a small, inhomogeneous environment, causing ultrasound waves to scatter. Through the DBI method, accurate determination of the object’s location and shape is achievable. The core challenge lies in the algorithm for precisely reconstructing the object. For the reasons mentioned above, during the development of the DBI method, model and parameter idealization was done with the aim of researching and developing an image reconstruction algorithm that surpasses traditional methods. Therefore, we modeled the object to be reconstructed as a simple cylindrical shape and the environment surrounding the object as homogeneous. The crucial task at hand is determining how to reconstruct an image that closely resembles the ideal objective function.

Assume that there are *N*_*t*_ × *N*_*r*_ transducers where *N*_*t*_ is the number of transmitters and *N*_*r*_ is the number of receivers (that gives *N*_*t*_ × *N*_*r*_ measurements). We need to reconstruct the target (grid area) which is divided into *N* pixels vertically and horizontally, i.e. *N*^2^ variables. Therefore, to be able to solve the system of equations conveniently, the number of measurements (*N*_*t*_ × *N*_*r*_) should be approximately or equal to the number of variables (*N*^2^). Therefore, depending on the actual resolution imaging requirements, the number of measurements also needs to be adjusted to be able to fully collect scattering information and restore the object accurately enough.

MATLAB is the numerical simulation application used, and it is run on a computer with a 2 GB of RAM and an intel core i3 CPU. Simulation parameters: Frequency *f*_1_ = 1 MHz, *f*_2_ = 2 MHz; *N*_1_ = 10; *N*_2_ = *N* = 20; transmitter number *N*_t_ = 8; receiver number *N*_r_ = 59; iteration number *N*_sum_ = 8; 7.3 mm is the scatter area diameter, 30% and 10% are sound-contrast and Gaussian noise, respectively. The transmitter and receiver distances to the object’s center are 50 mm and 60 mm, respectively. The frequencies used in this paper are based on prior work (*f*_1_ = 1 MHz and *f*_2_ = 2 MHz)^37^. The authors select little sound-contrast between 0.06% and 6%. Notwithstanding, this paper considers the harder issue (i.e., high sound-contrast). In this investigation, the excess phase indicator chosen is 8.76π (Δφ = 8.76π). The target is successfully recovered despite breaching the Born approximation condition and being influenced by the artifacts close to the goal’s center. Consequently, the Born approximation continues to provide a reliable solution. The phase from 0.004 to 16 is explored in the work^40^, and successfully reconstructed images are still obtained.

To ensure a good reconstruction of the objective function, the number of equations (or the number of measurements *N*_t_ × *N*_r_) must be equivalent to the number of variables (or number of pixels *N*^2^). Since *N*_t_ × *N*_r_ = 8 × 59 = 472, we choose the number of variables as ${N}^{2}={N}_{2}^{2}={20}^{2}=400$, meaning the number of measurements is 1.18 times larger than the number of variables. In this configuration, the amount of scattered information around the object is substantial, allowing for the computation and straightforward reconstruction of the ideal objective function as we employ the nearest-neighbor interpolation technique, the number of pixels in the vertical or horizontal direction before interpolation is chosen as *N*_1_ = 20/2 = 10. In fact, accurate object imaging depends on the correlation between the number of measurements (the product of the number of transmitters and receivers) and the number of pixels in the meshing area (the number of variables). In imaging to detect early objects causing breast cancer, the size of the measuring system is determined, so the number of probes that can be arranged on the measuring system can also reach a determined limit. With the need to create images with high resolution, the number of variables is much larger than the number of measurements. If we restore the object right in the first loop with a large number of variables, the estimation is quite less accurate than estimating with a small number of variables (small resolution). Therefore, starting images with a small resolution is necessary to be able to estimate and reach a certain convergence. In our simulation scenario, since the desired resolution is N_2_ = 20, the initial resolution is N_1_ = N_2_/2 = 10, the calculation results show quite good convergence in the first loop (as shown in Fig. 5) and the REE results in the last iteration are significantly better than the investigated methods. In fact, depending on the desired resolution to create the image (N_interest_), the starting resolution will change to N_interest_/2.

We carefully investigate the number of iterations for frequencies *f*_1_ (*N*_f1_) và *f*_2_ (*N*_f2_) to ensure that the image reconstruction performance achieves efficient quality when using the frequency-turning technique. Similarly, we thoroughly examine the number of iterations with low frequency and small resolution (*N*_Nf1_) and the number of iterations with high frequency and large resolution (*N*_Nf2_).

The DBI method is applied for detecting abnormalities in the breast, with the goal of reconstructing anomalies within the female breast. Consequently, the measurement configuration of the system is circular, and the transducers are arranged around the measurement system. To collect comprehensive information about the object, when one transmitter emits an ultrasound wave, the receivers pick up the scattered waves from anomalies at various angles around the object. In our simulation scenario, we use eight transmitters positioned at angles of 1^0^, 46^0^, 91^0^, 136^0^, 181^0^, 226^0^, 271^0^, and 316^0^, referencing some orientation. As the simulation scenario assumes a symmetric measurement system, the specific reference orientation is less crucial, and the focus is on scanning data from all angles around the object (360 degrees) to ensure gathering complete information for the most accurate image reconstruction.

In the bidirectional case, for the zero-order Bessel beam, the incidence pressure is

13 $${\overline{p}}^{inc}= {J}_{0}\left({k}_{0}\left|r-{r}_{k}\right|\right),$$

where ${J}_{0}$ and $\left|r-{r}_{k}\right|$ represent the zero-order Bessel function and how far the transmitter is from the ROI’s *k*^th^ point, respectively.

### Simulation results of the RT-DBI

**Figure 8:**
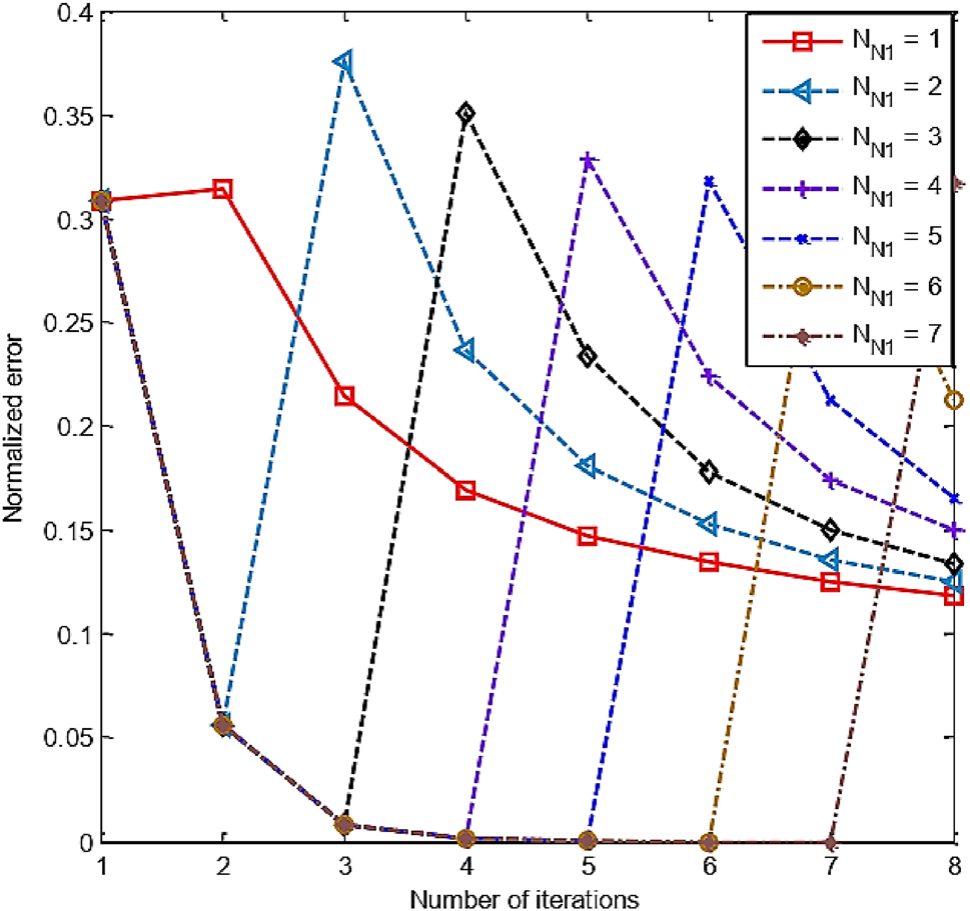
The RT-DBI’s normalized errors after iterations corresponding to various *N*_N1_s.

**Figure 9:**
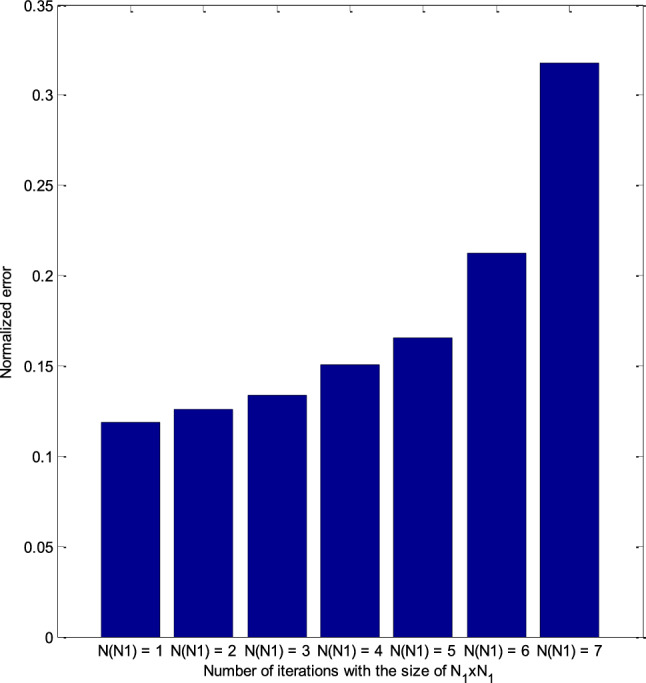
Comparison of RT-DBI methods’ normalized errors with the different number of iterations for the size of *N*_1_ × *N*_1_ (*N*_N1_).

Because *N*_sum_ = *N*_N1_ + *N*_N2_. Our goal needs to recover an image with a resolution of *N*_2_ × *N*_2_, i.e., related to the number of loops *N*_N2_. Therefore, the number of loops corresponding to resolution *N*_1_ × *N*_1_ is just a stepping stone to transition for *N*_N2_. If *N*_N1_ is too large, then *N*_N2_ may not be enough to restore the target image at *N*_2_ × *N*_2_ resolution to the desired quality. Figure 8 displays the RT-DBI’s normalized errors after iterations that correspond to various *N*_N1_s. It is clear that the best value of *N*_N1_ (i.e., *N*_N1_ = 1) offers the smallest error after *N*_sum_ iterations (see more in Fig. 9). Therefore, *N*_N1_ = 1 is chosen for further simulation.

### Simulation results of the FH-DBI

**Figure 10:**
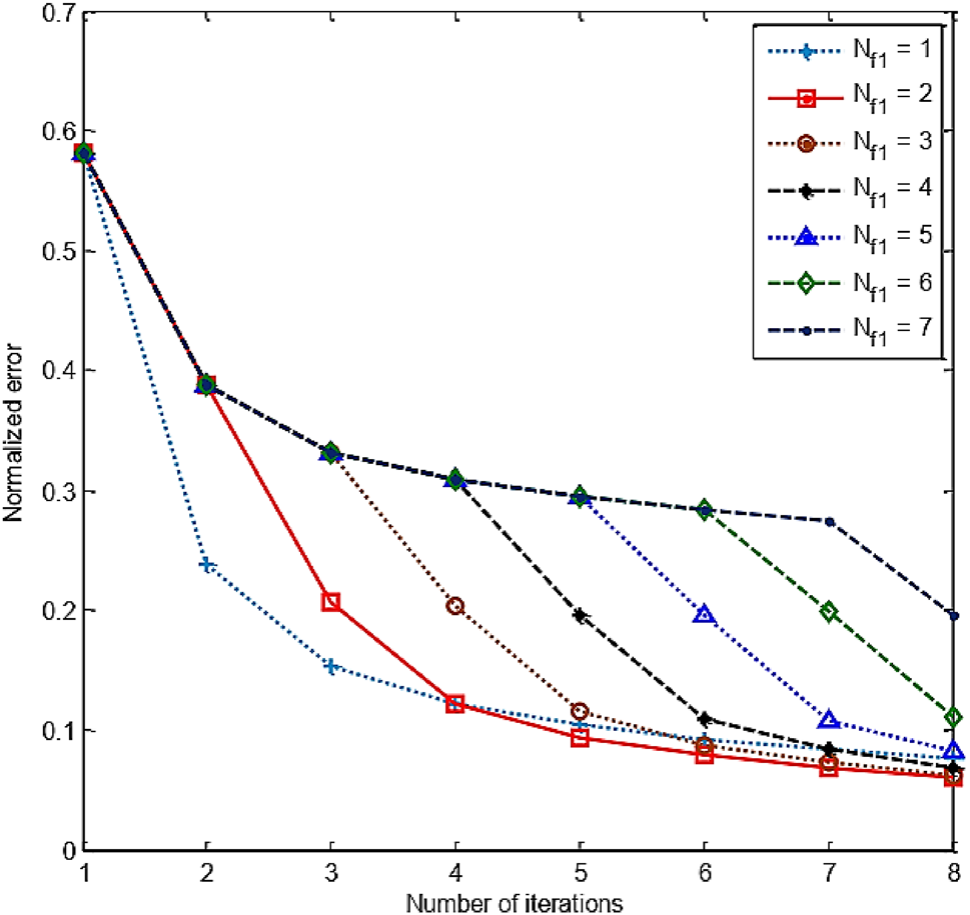
The FH-DBI’s normalized errors after iterations corresponding to various *N*_f1_s.

**Figure 11:**
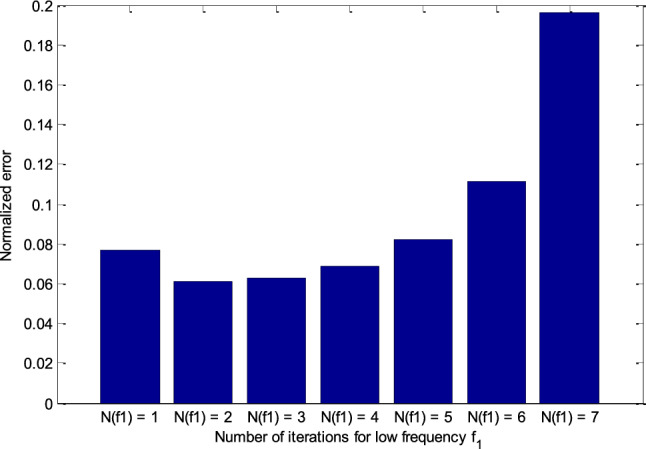
Comparison of FH-DBI methods’ normalized errors with the different number of iterations for low frequency *f*_1_ (*N*_f1_).

We fixed *N*_sum_ = *N*_f1_ + *N*_f2_. Suppose we need to recover an image with the desired contrast corresponding to the frequency *f*_2_ and the number of loops *N*_f2_. Thus, the number of loops corresponding to the frequency *f*_1_ (*N*_f1_) aims to estimate the target at a contrast close to the actual contrast of the object. Therefore, the first few loops will usually be for the *N*_f1_. The normalized errors of the FH-DBI over iterations correlated with various *N*_f1_s are shown in Fig. 10. It is demonstrated that the optimal value of *N*_f1_ (i.e., *N*_f1_ = 2) offers the smallest error after *N*_sum_ iterations (see more in Fig. 11). Therefore, *N*_f1_ = 2 is chosen for further simulation.

### Simulation results of the RT-FH-DBI

**Figure 12:**
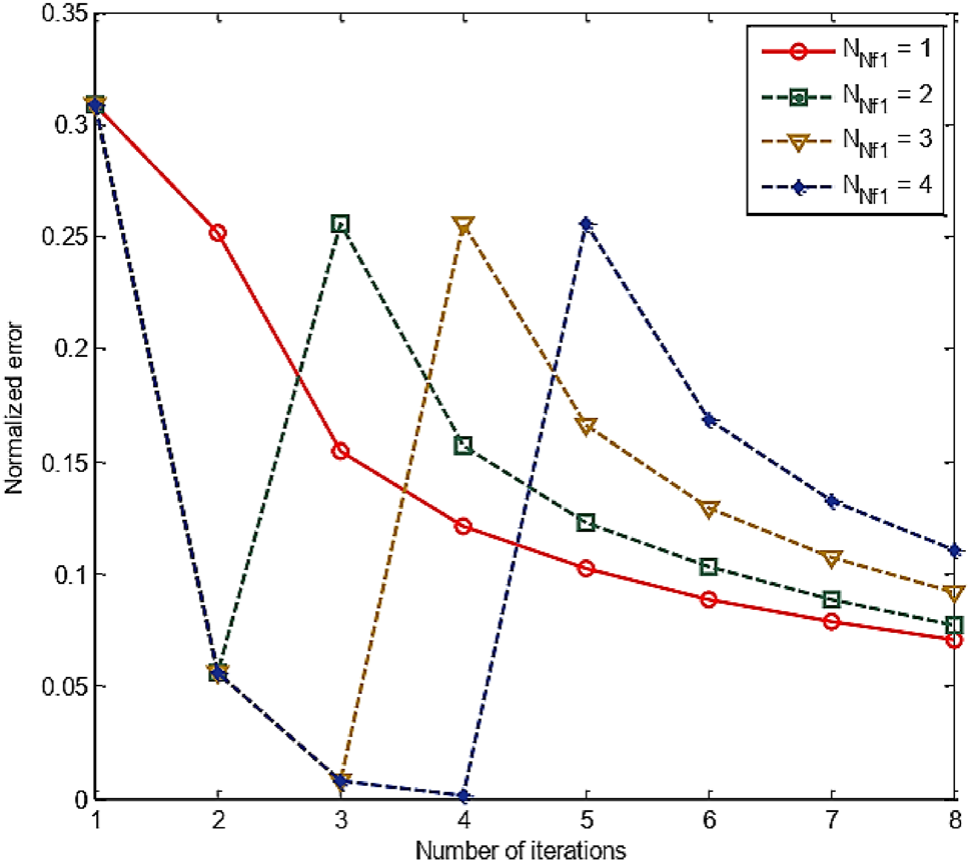
Normalized errors of the RT-FH-DBI-1 through iterations corresponding to different *N*_Nf1_s.

**Figure 13:**
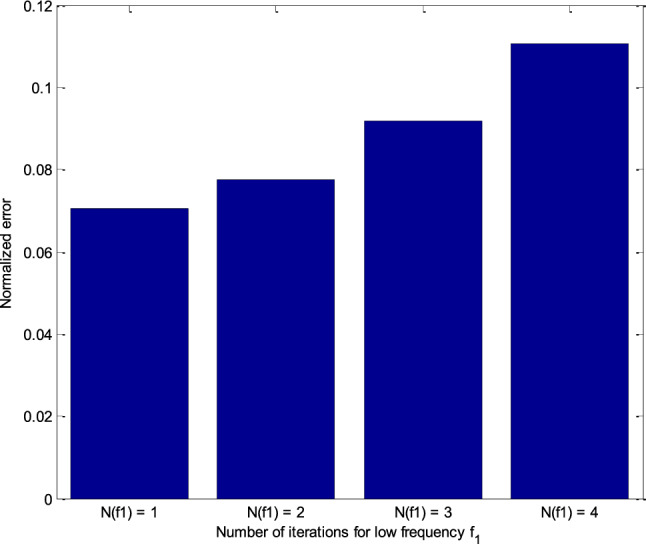
Comparison of RT-FH-DBI-1 methods’ normalized errors with the different number of iterations for low frequency *f*_1_ (*N*_Nf1_).

Simulation results of RT-FH-DBI-1 approach are shown in Fig. 12. The normalized errors of the first proposed approach are achieved through iterations corresponding to different *N*_Nf1_s. It is legibly that the best value of *N*_Nf1_ (i.e., *N*_Nf1_ = 1) offers the smallest error after *N*_sum_ iterations (see more in Fig. 13).

**Figure 14:**
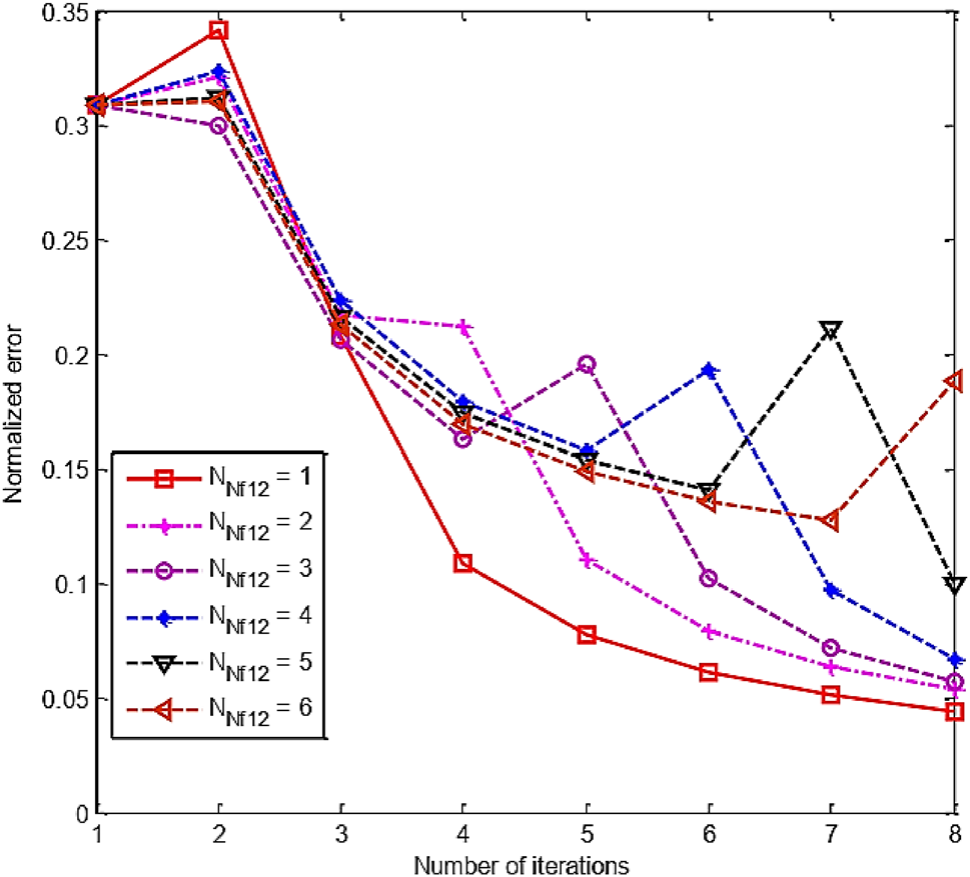
Normalized errors of the RT-FH-DBI-2 through iterations corresponding to different *N*_Nf12_s.

**Figure 15:**
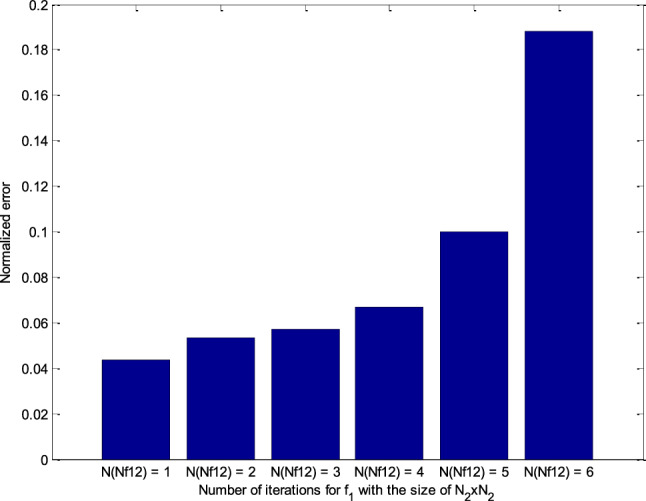
Comparison of RT-FH-DBI-2 methods’ normalized errors with the different number of iterations for *f*_1_ with the size of *N*_2_ × *N*_2_ (*N*_Nf12_).

Simulation results of RT-FH-DBI-2 approach are shown in Fig. 14. The normalized errors of the second proposed approach are achieved through iterations corresponding to different *N*_Nf12_s. It is distinctly that the best value of *N*_Nf12_ (i.e., *N*_Nf12_ = 1) offers the slightest error after *N*_sum_ iterations (see more in Fig. 15).

As shown in Figs. 12 and 14, the second suggested method’s normalized error (RRE = 0.0438) is less than the error of the first approach (RRE = 0.0704). Hence, the RT-FH-DBI-2 approach is chosen for further simulation.

**Figure 16:**
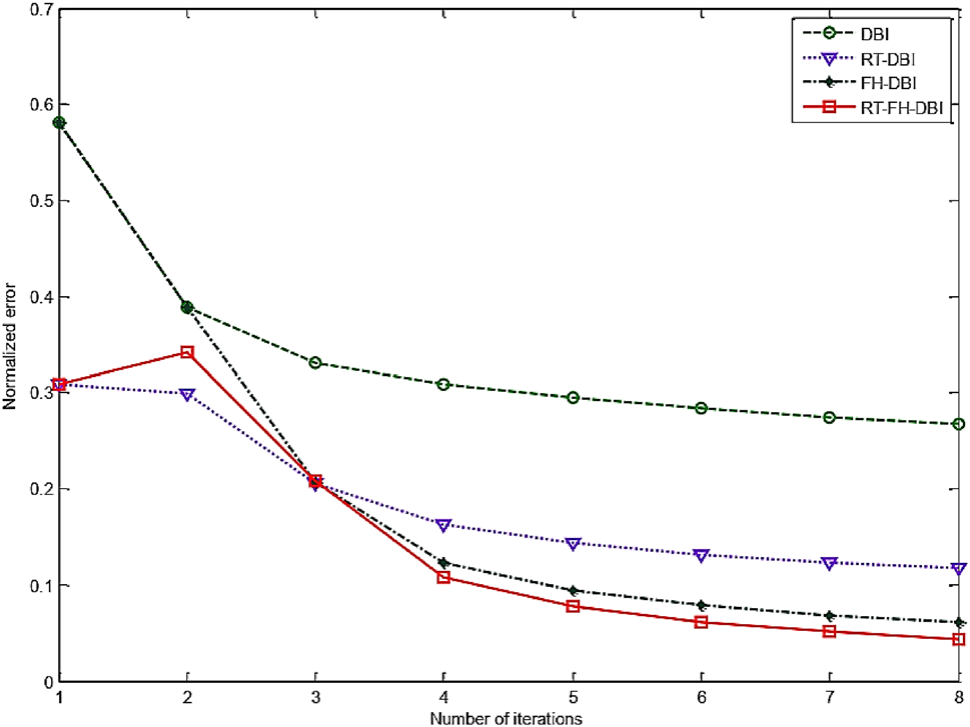
Normalized error comparison of different methods through iterations.

**Figure 17:**
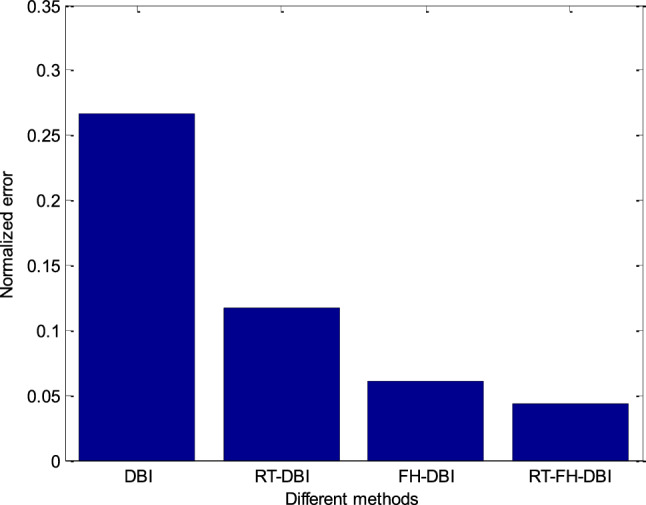
Comparison of different methods’ normalized errors after *N*_sum_ iterations.

The comparison of the error performance of the conventional DBI, RT-DBI, and FH-DBI methods with the RT-FH-DBI proposed method is presented in Fig. 16. It is clear that the standardized error of the RT-FH-DBI method is much lower than that of the DBI, RT-DBI, and FH-DBI methods (see more in Fig. 17).

**Figure 18:**
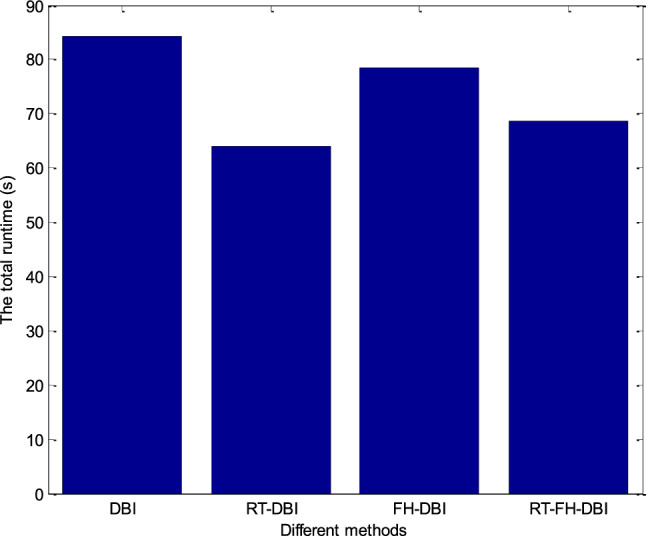
Following *N*_sum_ iterations, various methods are represented in the runtime totals chart.

Following *N*_sum_ iterations, Fig. 18 shows a chart of the overall runtime associated with different approaches. After the *N*_sum_ loops, the recovery times of the DBI, RT-DBI, FH-DBI, and RT-FH-DBI methods were 84.4, 64.1, 78.4, and 68.7 s, respectively. We see that the recovery time of the RT-FH-DBI method is reduced by 18.6% and 12.4% compared with DBI and FH-DBI methods. However, the recovery time of the proposed solution increased by 7.2% compared to the RT-DBI method. It is understandable because solutions that use interpolation (RT-DBI and RT-FH-DBI) will significantly reduce the recovery time. Between the two solutions, RT-DBI and RT-FH-DBI, the recovery time of RT-FH-DBI is slightly longer than that of RT-DBI, possibly due to the larger value of frequency *f*_2_ in the following loops; thus, the calculation will be longer.

**Figure 19:**
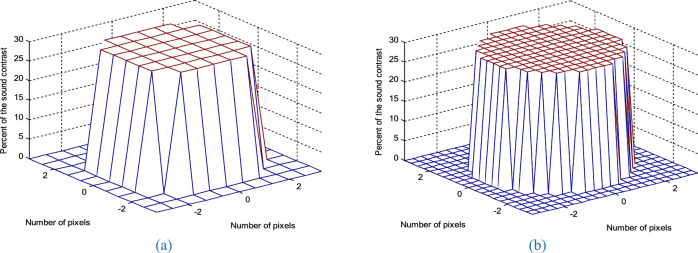
Ideal object function: (**a**) N = 10; (**b**) N = 20.

**Figure 20:**
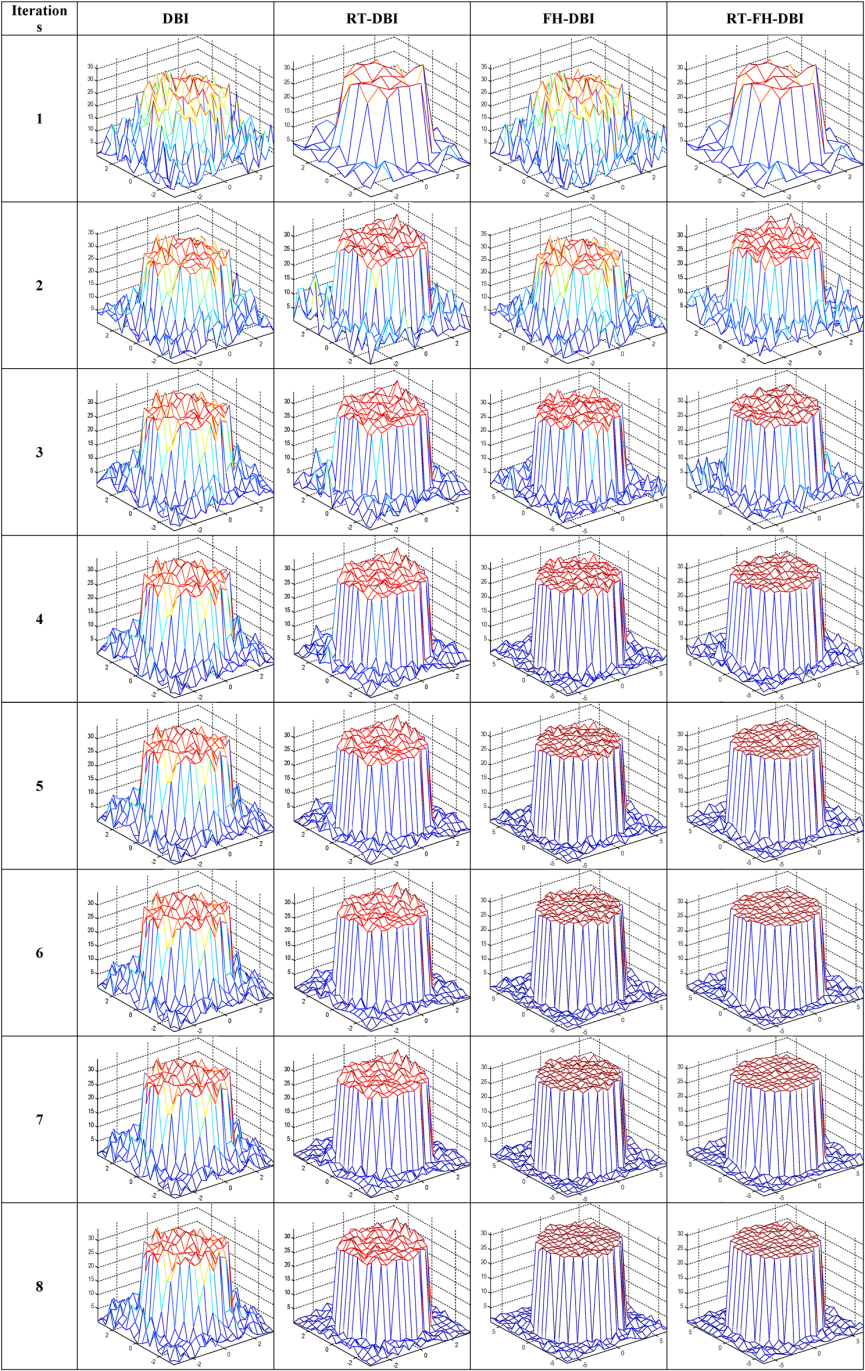
The reconstructed results of the different methods through iterations.

The true distribution of reconstructed objects which represent the actual spatial distribution of acoustic property (speed of sound) within the imaged medium are shown in Fig. 1 where the numbers of pixels along a certain axis are 10 (Fig. 19a) and 20 (Fig. 19b). The reconstructed images of the different methods through iterations are shown in Fig. 20. In the first loop, the object estimation with small resolution (*N*_1_ = 10) in the RT-DBI and RT-FH-DBI solutions, corresponding to low frequency (*f*_1_ = 1 MHz) is much better than the immediate estimation with large resolution (*N* = 20) for high frequency (*f*_2_ = 2 MHz) in DBI and FH-DBI solutions. In the last loop (*N*_sum_ = 8), it is easy to distinguish the recovered image quality between DBI, RT-DBI, and FH-DBI/RT-FH-DBI solutions. Visually, it will be difficult to distinguish the restored images between the FH-DBI and RT-FH-DBI solution. However, the RRE parameter in Fig. 16 shows that the RRE of the RT-FH-DBI is smaller than that of FH-DBI. It means that the image restored by the RT-FH-DBI solution offers the best recovery result of all the examined solutions.

**Figure 21:**
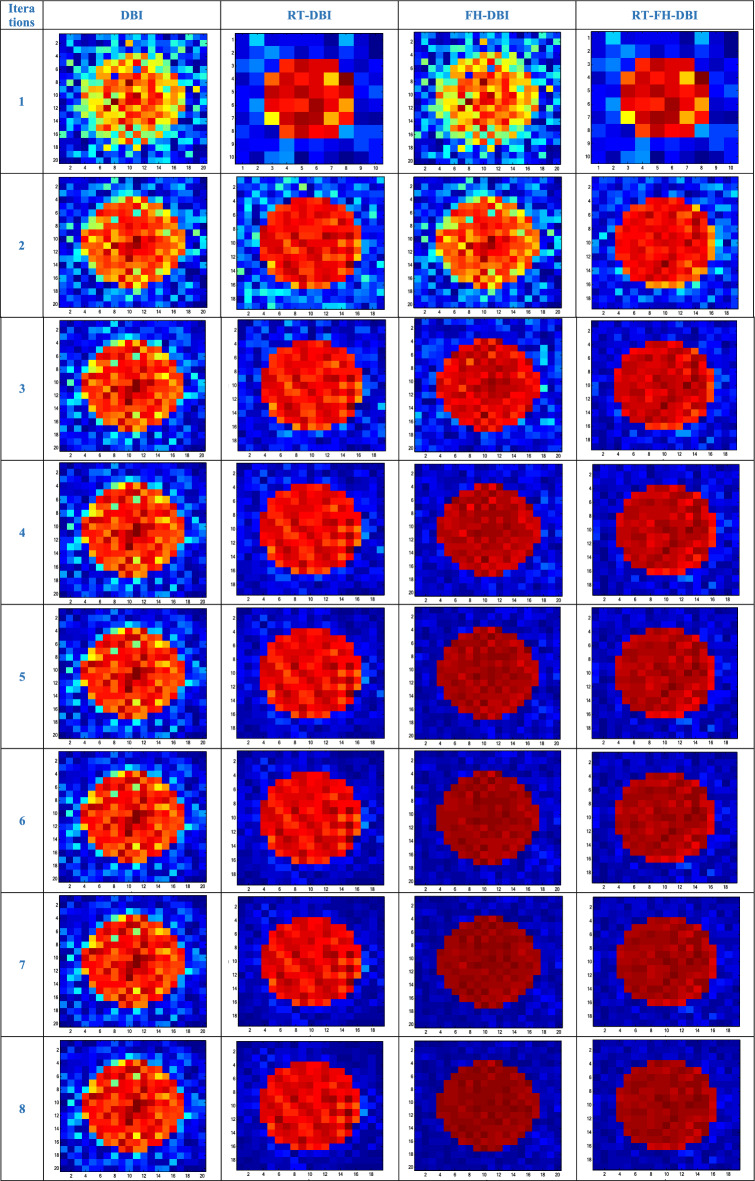
The reconstructed results of the different methods through iterations.

**Figure 22:**
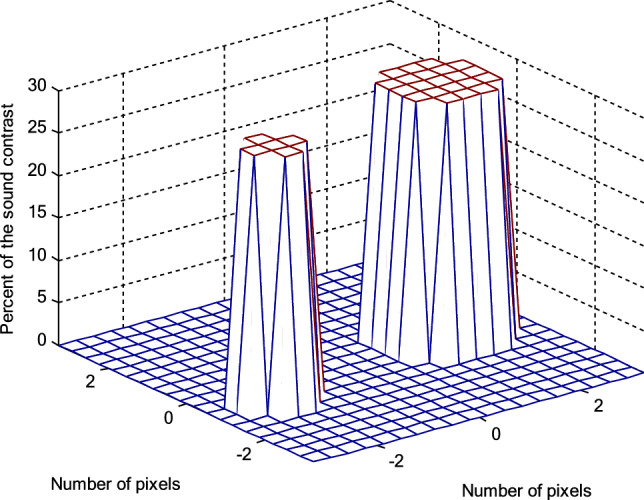
Ideal object function.

More experimental results of ideal object function which contains two objects in imaged medium are shown in Fig. 21, and the reconstructed images by the DBI, FH-DBI, RT-DBI, and RT-FH-DBI as shown in Figs. 22a–d respectively in case of number of measurements is 324 while number of variables is 400. That is, the number of measurements is 0.81 times the number of variables. The case where the number of measurements is smaller than the number of variables often happens in practice because we always want to create images with high resolution but are limited in the number of probes that can be arranged on a measuring system of a certain size. The normalized errors after the last iteration of DBI, FH-DBI, RT-DBI, and RT-FH-DBI methods are 1.2374, 0.4582, 0.2940, and 0.2550, respectively. The DBI has the highest normalized error at 1.2374, indicating it is the least accurate method among the four. The FH-DBI significantly reduces the error, followed by RT-DBI, which further improves the accuracy. The RT-FH-DBI achieves the lowest normalized error at 0.2550, making it the most accurate method among the ones compared.

## Discussion

Within the framework of the DBI method, the precise determination of pixel values in the region of interest is achieved. In the presence of a slightly non-uniform medium, the propagation of ultrasonic waves leads to scattering phenomena, from which valuable scattering data is acquired. The DBI method plays a pivotal role in accurately ascertaining the spatial position and morphology of objects. It is evident that the central challenge lies in developing an algorithm capable of precisely reconstructing objects with exceptional performance. Through the utilization of ultrasound tomography, any heterogeneous environment can be effectively visualized, provided the contrast is sufficiently high. Consequently, to foster advancements beyond the conventional DBI method, our research and development efforts involve the idealization of models and parameters in constructing a DBI simulation. In pursuit of achieving a more refined image recovery algorithm, the simulated object for restoration is simplified to a circular cylinder, assuming a homogeneous environment devoid of complicating factors such as mammary glands. The primary objective remains to attain image recovery that closely approximates the ideal objective function. When using the iteration method to restore an image, we have two methods to exit the iteration. Firstly, we fixed the required number of iterations *N*_sum_ and the image creation process will work until *N*_sum_ stops. Second, we set a certain threshold that the image quality of the system needs to be achieved, the image creation process will continue until the image quality reaches that threshold, then stop. However, in this solution, it is possible that the method in use needs a small number of loops that have reached the threshold, it may also be a lot of loops or it may never stop depending on the convergence rate of this approach. Therefore, in this work, we choose the solution to fix the quantity of iterations to observe the convergence speed after the *N*_sum_ loops.

**Figure 23:**
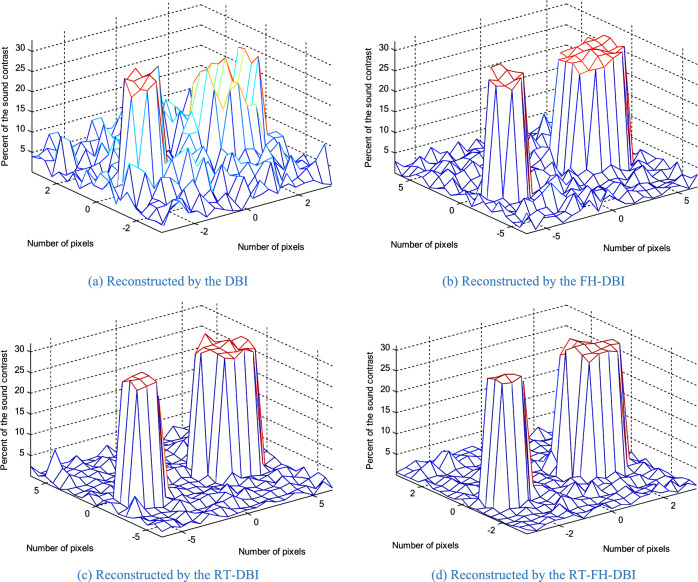
Reconstructed images by different approaches.

The approach of merging two frequencies can benefit from both lower and higher frequencies, as can be observed. It has a higher convergence rate and lower error. As we analyze in this paper, the interpolation method also affects the imaging quality. The proposed method RT-FH-DBI exploited both the advantage of combining two frequencies and the interpolation technique. The simulation results proved that RT-FH-DBI could improve the image’s resolution. Figures 20 and 23 further demonstrated that multi-frequency DBI image reconstruction outperforms standard DBI and the findings of our earlier research^34^. The broad frequency bandwidth of the ultrasonic probes allows for the feasibility of employing a multi-frequency technique. In order to achieve convergence in our study, we establish a relatively low value for the starting frequency (*f*_1_). After that, we use the second frequency (*f*_2_ = 2*f*_1_) to save the high spatial resolution. It is worth mentioning that the anticipated images in Figs. 20, 21 and 23 include a lot of interesting properties. It significantly supports the efficacy of RT-FH-DBI in conjunction with RT-DBI and FH-DBI.

The key innovation of combining frequency hopping and resolution tuning in ultrasound tomography lies in the holistic enhancement of imaging capabilities. This approach addresses the limitations of existing methods by integrating the strengths of both techniques, leading to improved penetration, resolution, adaptability, and overall image quality. Frequency hopping involves varying the ultrasound frequencies used during the imaging process. Different frequencies provide different penetration depths and resolutions, which helps in capturing a more comprehensive image of the tissue. While resolution tuning adjusts the resolution dynamically during the iterative reconstruction process allows for capturing both coarse and fine details effectively. By integrating frequency hopping with resolution tuning^33,34^, the method leverages the strengths of both approaches, leading to improved image quality and detail resolution. Indeed, when comparing the RT-FH-DBI algorithm with frequency hopping^36,37^ and resolution tuning, the results show that after the last iteration the RT-DBI has the highest normalization error (0.1171), indicating it is the least accurate and effective among the three methods; the FH-DBI has a lower normalization error (0.0611) than RT-DBI, showing better performance; the RT-FH-DBI has the lowest normalization error (0.0438), demonstrating the best performance in terms of accuracy and convergence.

While our research yielded significant results, certain limitations exist in the study itself. Firstly, it can be observed from Fig. 16 that the normalized error of RT-FH-DBI stabilizes after the 6^th^ iteration, reaching a minimum value. By lowering the acceptable reconstruction error in the reconstructed algorithm, this floor can be lowered. This will result in a more difficult computing procedure, as a result, a significantly extended imaging time. Second, the sole focus of this study is numerical simulation. Numerous factors, including the number of transceivers, meshing area, noise level, number of repeats, and frequency, may have an impact on the quality of the image reproduction^41^. The way the constraints are applied to the simulation scenarios affects the calculation cost. Third, the Born approximation is widely used and generally reliable in solving certain types of imaging problems, it has limitations that can be exacerbated by noise and artificial interference. However, in our work, to mitigate these issues, we utilized: (a) Regularization method, namely Tikhonov regularization, is used to stabilize the solution; (b) Using multi-frequency information is indeed a valuable strategy to overcome interference and improve the accuracy and stability of imaging, especially when using methods like the Born approximation. Noise and interference typically vary with frequency. By collecting data at multiple frequencies, it is possible to average out the noise effects. This averaging process can help to isolate the true signal from the noise, leading to a clearer and more stable image. Furthermore, different frequencies interact with the medium in different ways. Some frequencies might be more affected by certain types of noise or interference than others. Multi-frequency methods can exploit this diversity to mitigate the impact of frequency-specific interference. In practice, implementing multi-frequency approaches involves collecting data at various frequencies and integrating this information during the imaging process. This can be done using advanced signal processing techniques and inversion algorithms designed to handle multi-frequency data. The result is a more accurate, stable, and reliable imaging solution that is less susceptible to the adverse effects of noise and artificial interference.

## Conclusion

Backscattering using the DBI, in contrast to traditional ultrasound imaging using echolocation, is a typical technique that may be used to discern features smaller than the incident wave’s wavelength. This paper has successfully applied frequency-hopping (dual frequency) and resolution-turning (nearest-neighbor interpolation) techniques in the DBI to speed up sound-contrast recovery and improve image recovery quality. This method also provides a straightforward installation compared to other methods. Therefore, it can avoid many types of errors in measurement. To demonstrate the effectiveness of this strategy, simulation scenarios of sound contrast recovery were executed. By using 3D recovery and experimental testing, this work can be improved.

### Supplementary Information

Supplementary Information.

## Data Availability

Data is provided within supplementary information files.
